# Blood Loss and Transfusion Rates in Microsurgical Head and Neck Reconstruction

**DOI:** 10.1097/GOX.0000000000001988

**Published:** 2018-11-07

**Authors:** Anson Nguyen, Hope Shin, Michel Saint-Cyr, Charles Verheyden

**Affiliations:** From the Division of Plastic Surgery, Department of Surgery, Baylor Scott and White Health, Temple, Tex.

## Abstract

**Background:**

Free flap reconstruction cases of the head and neck are often complex, long and have a multitude of risks. One of the greatest risks is intraoperative blood loss and need for transfusion. The purpose of this study was to examine basic patient and procedure characteristics in head and neck free flap reconstruction pre- and postoperatively that may help to predict severity of blood loss.

**Methods:**

A retrospective chart analysis of 67 free flap reconstructions for head and neck defects was performed. Patient characteristics, surgical variables, length of stay, and postoperative complications were reviewed and compared between the transfused and nontransfused patients. Characteristics between transfused and nontransfused patients were analyzed using two-tailed *t* tests and Fisher’s exact tests.

**Results:**

Of the 67 procedures, 19 reconstructions (28.4%) required a transfusion. Transfused patients were found to have a lower preoperative hemoglobin and elevated coagulation labs. The average length of stay was also statistically longer for transfused patients. There was no statistical difference in patient characteristics, length of surgery, type of free flap, or complication rate in the transfused versus nontransfused patients.

**Conclusions:**

Our study demonstrates that head and neck microsurgical resection and reconstruction presents patients with a transfusion risk of over 28%. We found that patients with a lower preoperative hemoglobin and abnormal coagulation levels are at a higher risk for receiving a transfusion. We also have demonstrated that patients who received a transfusion had a statistically significant longer length of stay.

## INTRODUCTION

Head and neck cancer accounts for more than 550,000 newly diagnosed cases in the world each year.^1^ In recent years, due to technological advancement, better imaging, and advances in surgical technique,^2,3^ the management of head and neck and cancer has evolved and improved. However, inherent to microsurgical reconstruction of head and neck defects is the challenge that it presents not only to the surgeon but also to the patient as well. Often cases are complex and prolonged, performed in conjunction with other specialties, and often result in significant morbidity. The hurdles that these patients must overcome to convalesce are not trivial.

One of the most common and obvious risks in surgery and particularly within this field is intraoperative blood loss. With meticulous care in maintaining hemostasis through the use of clips, cautery, and suture ties, the risk of significant bleeding can be reduced but never truly eradicated. A multitude of studies and guidelines pertaining to blood transfusions are available but because substantial variability persists with regard to patient population and outcomes, it is difficult to draw an applicable conclusion from many of these reports. As a result of this, standard protocols for blood transfusion vary dramatically from one institution to another.

Within plastic surgery, very few studies have been conducted regarding the predictability of intraoperative blood loss. This may in part stem from the fact that plastic surgery as a whole is an extremely diverse specialty and the spectrum of procedures can range from simple local flaps to major multistage reconstructive cases. However, hemodynamic stability is imperative to the success of any operation. Without adequate perfusion, there is a threat of flap failure, graft loss, and wound healing complications in addition to other systemic health consequences. Head and neck microsurgical reconstruction cases are noteworthy for potential blood loss since these are lengthier and more complex procedures and are more likely to receive a blood transfusion.

The purpose of this study was to examine basic patient and procedure characteristics in head and neck free flap reconstruction pre- and postoperatively that may help to predict severity of blood loss. By examining this, we may be able to stratify a risk assessment for operations to be conducted. By identifying such characteristics or risk factors for transfusions, we may be able to proactively intervene and mitigate the consequences of delayed identification of the need for blood transfusion.

## METHODS

This was a retrospective study at a single institution between the years of 2010 and 2016 with the approval of the international review board. The CPT codes that were included were 15756, 15757, 15758, and 20955. Inclusion criteria were all patients who underwent microsurgical free flap reconstruction of head and neck defects. To reduce confounding variables, patients were excluded if they experienced significant bleeding secondary to injury or surgery before plastic surgery intervention during the same hospitalization. A total of 64 patients and 67 reconstructive procedures met criteria. Data were reviewed and compiled into a database including patient characteristics such as age, body mass index (BMI), and comorbidities of hypertension and diabetes. Other variables were also compiled, including the type of free flap, preoperative coagulation labs, duration of surgery, and change in hemoglobin. The main outcome measured was the need for transfusion during surgery and within 2 days postoperatively. The threshold for the need for transfusion was a hemoglobin less than 7.0 g/dl.

In addition to the above variables, postoperative complications and length of stay were compared between the transfused and nontransfused patients. Complications were categorized as major or minor. A major complication was one that required a reoperation or resulted in flap loss, and a minor complication included less significant intervention but was still detrimental to the flap, donor site, or patient. Characteristics between transfused and nontransfused patients were analyzed using two-tailed *t* tests and Fisher’s exact tests. Statistical analysis was performed using GraphPad Prism (GraphPad Software, Inc., San Diego, Calif.), and significance was defined as a *P* value less than 0.05.

## RESULTS

A total of 67 procedures on 64 patients were included in this study during the years of 2010 to 2016 at Baylor Scott & White. Of the 67 procedures, 19 reconstructions (28.4%) required a transfusion.

There were 47 male and 18 female patients included, with the ages ranging from 34 to 82 years. The median age was 61 years, and the mean age was 59.7 years. Out of the 67 procedures, 62 procedures (92.5%) were for malignancies of the head and neck and 5 (7.5%) were for osteoradionecrosis. The types of free flaps performed are summarized in Table 1.

**Table 1.**
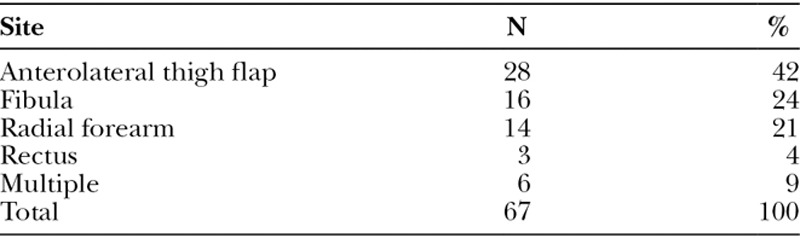
Donor Sites

Preoperative hemoglobin, PT, INR, and the change in preoperative to postoperative hemoglobin were all found to be statistically significant between the transfused and nontransfused patients (Table 2). Preoperative hemoglobin in patients requiring transfusion was 11.15 g/dL compared with 12.83 g/dL in those who did not require a transfusion (*P* = 0.001). Preoperative to postoperative change in hemoglobin was also found to be of significant difference between transfused (-2.93) and nontransfused patients (-2.18; *P* = 0.02). PT and INR were also found to be statistically higher for transfused patients. These results are found in Table 2.

**Table 2.**
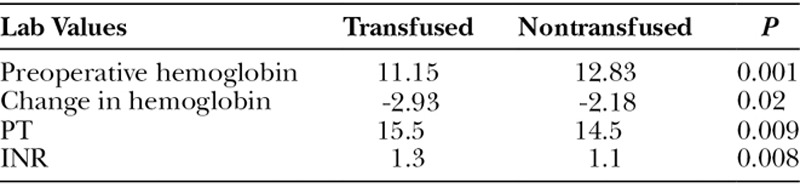
Preoperative Laboratory Values

After reviewing basic patient characteristics including patient age, BMI, and comorbidities of diabetes and hypertension, there was no statistically significant difference found between the transfused and nontransfused patients. These results are summarized in Table 3.

**Table 3.**
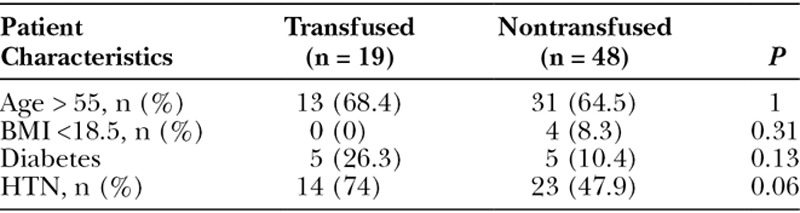
Patient Characteristics

**Fig. 1.**
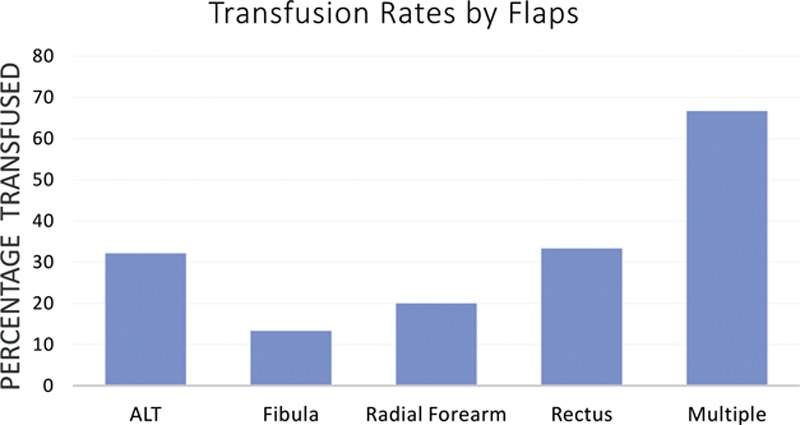
Percentage transfused by composition of flaps.

**Fig. 2.**
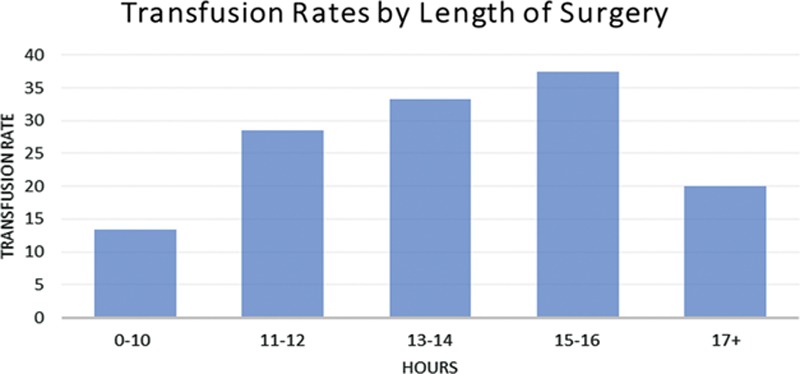
Transfusion rates by length of surgery.

When reconstruction was analyzed by flap type, no significant difference in transfusion rates was noted between ALT, fibula, radial forearm, or rectus abdominis flaps (*P* = 0.15). This was summarized in Figure 1. Length of surgery’s impact on blood loss was analyzed by dividing the surgery length into 6 categories: 0–10, 11–12, 13–14, 15–16, and 17+ hours. However, the length of surgery did not have a significant effect on transfusion rate with a *P* value of 0.65 (Fig. 2).

The average length of stay and complication rates were also compared between the transfused and nontransfused group. The average length of stay for the transfused group was 16.2 days, whereas the nontransfused group had an average stay of 12.1 days (*P* = 0.03). The transfused group had a major complication rate of 14.3% and the nontransfused group had a major complication rate of 8.3%. However, the overall complication rates between the transfused and nontransfused groups were found to not be statistically significant with a *P* value of 0.87 (Table 4).

**Table 4.**
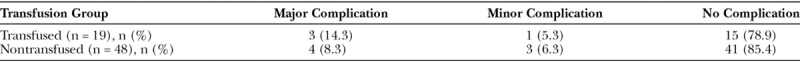
Complication Rates

## DISCUSSION

In this study, we reviewed 67 head and neck free flaps and the association between basic patient and procedure variables and the need for transfusion. We also looked at whether a transfusion had any association with increased length of stay and complication rate. The overall transfusion rate in our study was 28.4%, which is comparable with previous literature.^4^

After comparing preoperative basic patient and procedure characteristics between the transfused and nontransfused patients, lower preoperative hemoglobin was significantly associated with a need for a transfusion. In another study by Shah et al.^5^, low preoperative hemoglobin level was also the strongest independent predictor of blood transfusion. However, in our study, we found that higher intraoperative change in hemoglobin, PT, and INR were also significant predictors of a need for transfusion. In the patients who required a transfusion, our hypothesis is that the elevated preoperative PT and INR leads to poor clotting ability and ultimately increased intraoperative bleeding.

In our study, we did not find any statistical difference in preoperative basic patient characteristics, duration of surgery, or composition of the free flap in the transfused and nontransfused patients. We looked at basic characteristics such as age, BMI, and comorbidities including hypertension and diabetes and there was no association. Types of flaps included free fibula, ALT, radial forearm, rectus, and multiple flaps, and this was not a predictor of transfusion rates. There was also no association found in length of surgery and rates of transfusion. In previous literature, preoperative factors including an underweight BMI, osseous free flap reconstruction, and low preoperative hemoglobin level were shown to be independent predictors of a need for transfusion.^3^ Differences in our findings regarding BMI could be due to the fact that most the patients in our study were normal weight or overweight, and only 6% of total patients had a BMI < 18.5. We did not find any difference in transfusion rate for osseous flaps, but that may a result of not independently comparing multiple flaps instead of grouping them into a “multiple” category in our analysis.

Our results demonstrated a significant increased length of stay for patients who received a transfusion, with the average length of stay 16.2 days compared with 12.1 days for patients who did not receive a transfusion. However, we did not find a statistically significant difference in the minor or major complications in the transfused versus the nontransfused group of patients. In previous studies, low preoperative hemoglobin was a significant predictor for both surgical complications and hospital stay in head and neck reconstruction^6^, and perioperative complications were significantly associated with intraoperative blood loss^7^.

Based on the findings from our study, several factors may help to potentially reduce the severity of blood loss and transfusion rates from head and neck reconstructive surgery. We recommend measuring hemoglobin levels at least 30 days before surgery and optimizing patient’s hemoglobin levels before surgery. Several studies have shown that taking iron, folic acid, and vitamin B12 can stimulate erythropoiesis if given enough time before surgery. It is recommended that adults receive 150–200 mg/d of iron in deficient states. Reticulocytosis should be seen in 7–10 days with a hemoglobin level increase of 1 g/dL every 2–3 weeks. Anemia due to folate or vitamin B12 deficiency can also be treated with 1 mg of folic acid daily or intramuscular cobalamin injections weekly. Reticulocytosis can be expected in 3–5 days with an increase in hemoglobin within 10 days.^8^ Furthermore, patients with lower hemoglobin levels may benefit from preoperative type and cross due to higher likelihood for transfusion while those with adequate blood levels may not warrant extra testing.

Our study is not without limitations. Due to the retrospective design of our study, our analysis is limited to the information in the medical chart. In addition, our study also has a relatively small sample size with 64 patients, which may limit the generalizability of our study. Another limitation includes the heterogenicity of patients that require head and neck reconstruction. The size of the defect and the flap used can vary from patient to patient, which could also limit the generalizability of our results. In addition, the amount of blood loss during the resection part of the operation can be variable and dependent on another surgical team, and this often cannot be controlled.

In conclusion, our study demonstrates that head and neck microsurgical resection and reconstruction presents patients with a transfusion risk of over 28%. Our results indicated that patients with a low preoperative hemoglobin and abnormal coagulation levels are at a higher risk for receiving a transfusion. We also have demonstrated that patients who received a transfusion had a statistically significant longer length of stay. By identifying patients who are at higher risk for transfusions, prehabilitation and optimizing the patient’s condition before surgery can potentially decrease transfusion rates in head and neck reconstructive surgery.
